# Supplementary material for: Learning cis-regulatory principles of ADAR-based RNA editing from CRISPR-mediated mutagenesis
Source: Nat Commun. 2021 Apr 12;12:2165. doi: 10.1038/s41467-021-22489-2 (PMC8041805; doi:10.1038/s41467-021-22489-2)
Supplement: Supplementary file 1 — Supplementary Information [file 41467_2021_22489_MOESM1_ESM.pdf]

Supplementary Fig. 1

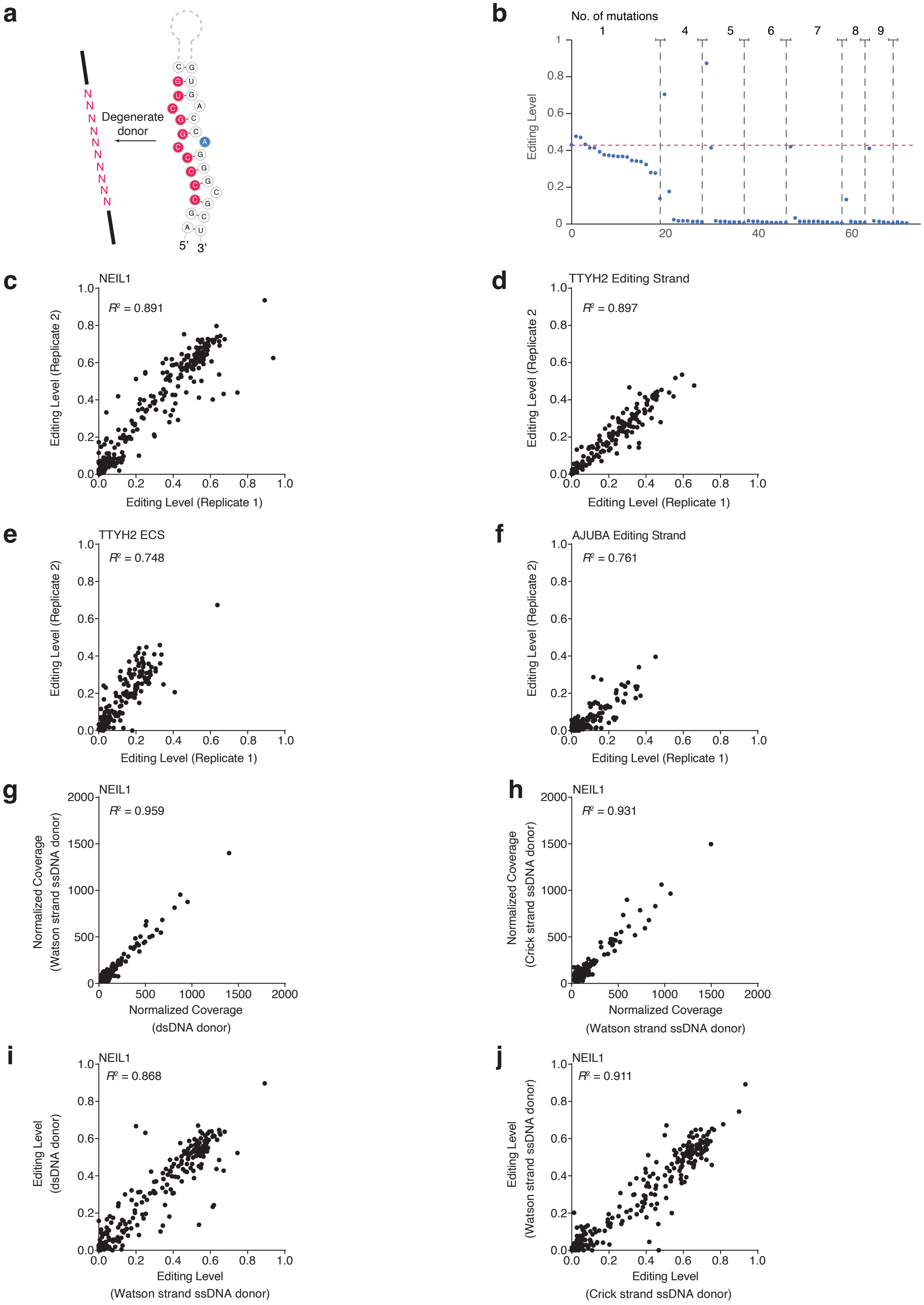

Supplementary Fig. 1 Reproducibility of targeted-mutagenesis and RNA editing measurements. a Degenerate donor oligos are designed for a 10 nt region of the ECS in the TTYH2 substrate. The mutagenized

region is highlighted in red and the editing site in blue. **b** The distribution of editing level by the number of mutations from the results of the degenerate TTYH2-ECS library from **a**. **c** Two biological replicates for NEIL1. **d** Two biological replicates with variants in the TTYH2 edit strand. **e** Two biological replicates with variants in the TTYH2 ECS. **f** Two biological replicates of variants in the AJUBA edit strand. **g,h** Comparing different types of donor oligos for CRISPR knock-in efficiency of NEIL1 variants. **i,j** Editing level measurement reproducibility using different versions of NEIL1 donor oligos (ssDNA vs dsDNA).

Supplementary Fig. 2

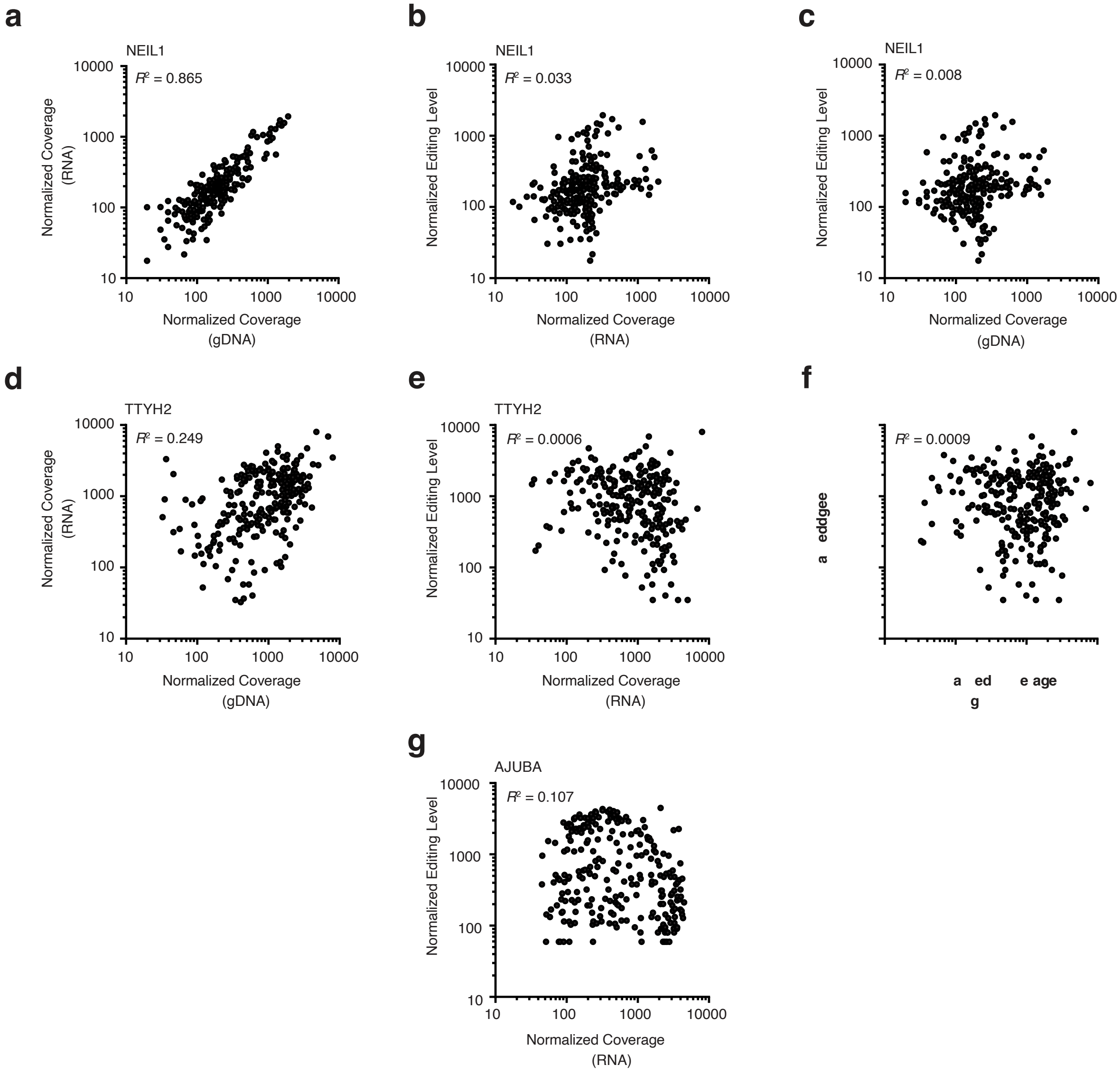

Supplementary Fig. 2 Comparison of RNA coverage, gDNA coverage and Editing Levels. a,b,c NEIL1. **d,e,f** TTYH2. **g** AJUBA. The outlier of the RNA coverage and gDNA coverage data were removed by percent cumulative density function to remove the data points larger than the 99% distribution. The RNA coverage, gDNA coverage and editing level are quantile normalized for pair-wise comparison.

Supplementary Fig. 3

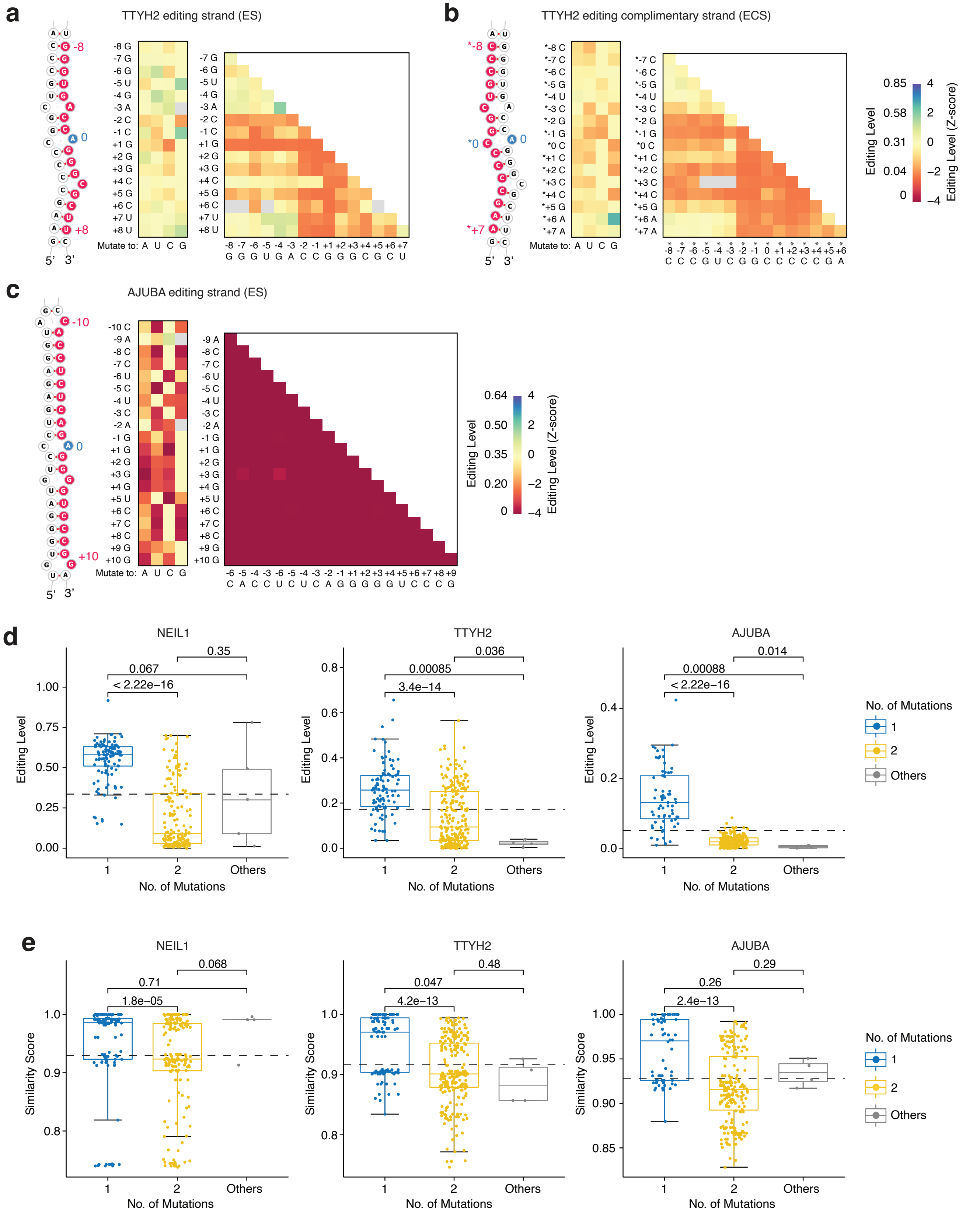

Supplementary Fig. 3 Editing levels from targeted-mutagenesis libraries. a Heatmap of editing levels from single- and double-mutations in the editing strand of TTYH2. **b** Heatmap of editing levels from single mutations in the editing complementary sequence (ECS) of TTYH2. Editing level of WT TTYH2 is 0.31. **c** Heatmap of

editing levels from single- and double-mutations in the editing strand of AJUBA. The Z-score is calculated for each RNA library as described in Methods and the WT editing level Z-score is 0. **a** to **c** heatmaps share the same color scale. **d** Comparing editing levels of variants with the mutation type. **e** Comparing the normalized similarity score of the variants with mutation type. In **d** and **e,** single-mutation (blue dot), double-mutation (yellow dot), others (gray dot) refer to mutation types other than single or double mutations, such as indels or multiple mutations. In **d** and **e,** two-sided Wilcoxon rank-sum test was utilized, and *P* value are labeled on the plot. Boxplots in **d** and **e** are presented as median-centered, with the box bounds indicating the 75 percent interquartile range (IQR). Whiskers extend to the data points that fall within the median ± 1.5*IQR. The data points shown for NEIL1 are the average editing level from six biological replicates and for TTYH2 and AJUBA from two biological replicates

Supplementary Fig. 4

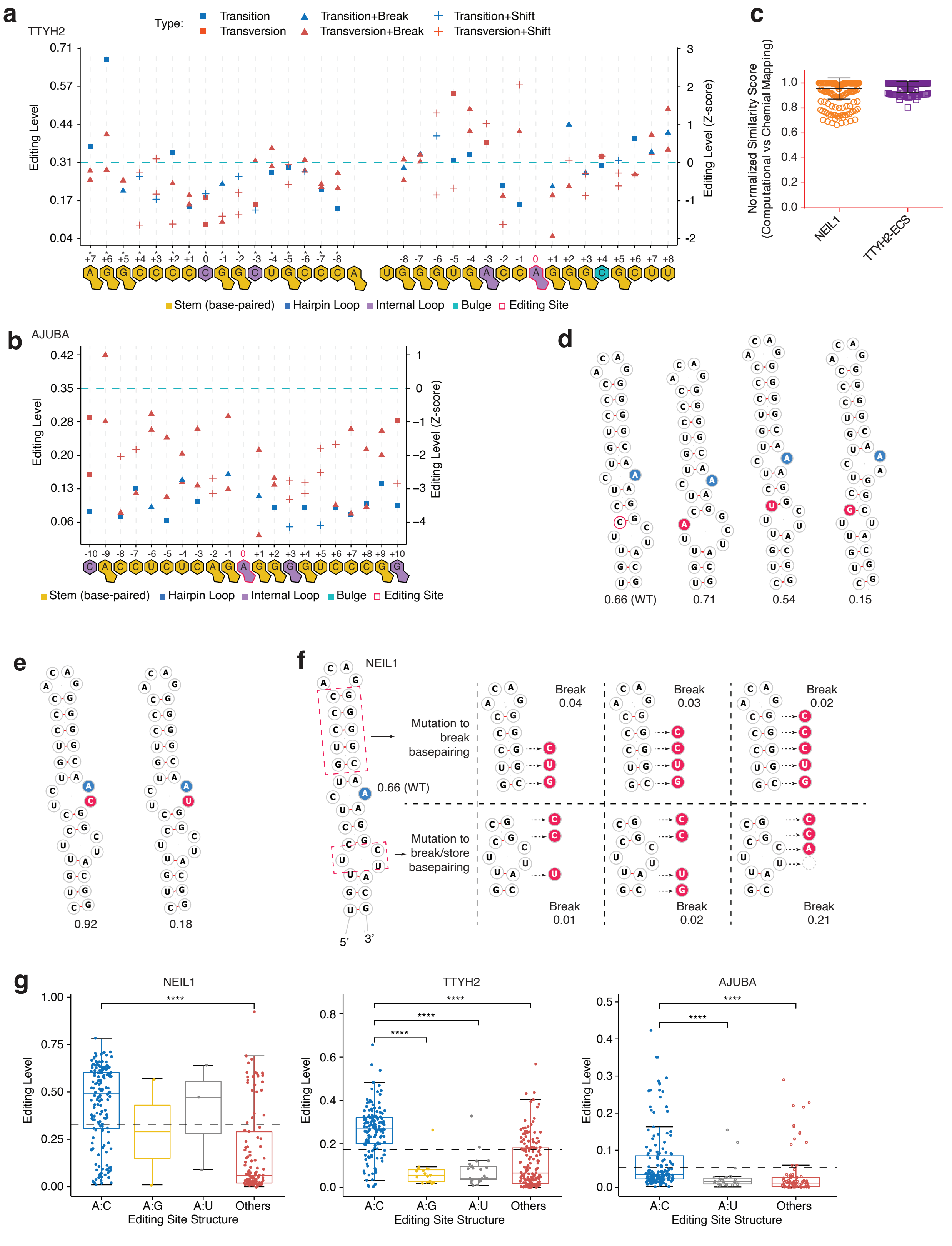

Supplementary Fig. 4 Selected examples of variants of NEIL1, TTYH2 and AJUBA. a,b Position-specific effects of **a** TTYH2 and **b** AJUBA single mutations categorized by six types: transition mutation that does not affect RNA secondary structure (Transition, blue square), transition mutation that disrupt the base pair at the

mutation site (Transition + Break, blue triangle), transition mutation that leads to disruption of more than one base pair and/or formation of new base pair (Transition + Shift, blue cross), transversion mutation that does not affect RNA secondary structure (Transversion, red square), transversion mutation that disrupt the base pair at the mutation site (Transversion + Break, red triangle), transversion mutation that leads to disruption of more than one base pair and/or formation of new base pair (Transversion + Shift, red cross). **c** Comparison of the computationally predicted and experimentally inferred MFE structure of NEL1 and TTYH2-ECS. A normalized similarity score means identical structures. **d,e** Single-mutations at different locations lead to different editing levels. **f** Effects of compound mutations that break the base pairing in the 5' and 3' structure of NEIL1 (WT NEIL1 editing level is 0.66). **g** Comparing the editing levels of variants with different editing site structure. "Others" means the editing site A is located in a structure other than the 1:1 mismatch internal loop. The A:A mismatch was not presented in our RNA library. Boxplot: center line, median; box limits, upper and lower quantiles; whiskers, 1.5x interquartile range. *P* values from two-sided Wilcoxon rank-sum test are labeled for each test set. The data points shown for NEIL1 are the average editing level from six biological replicates and for TTYH2 and AJUBA from two biological replicates

Supplementary Fig. 5

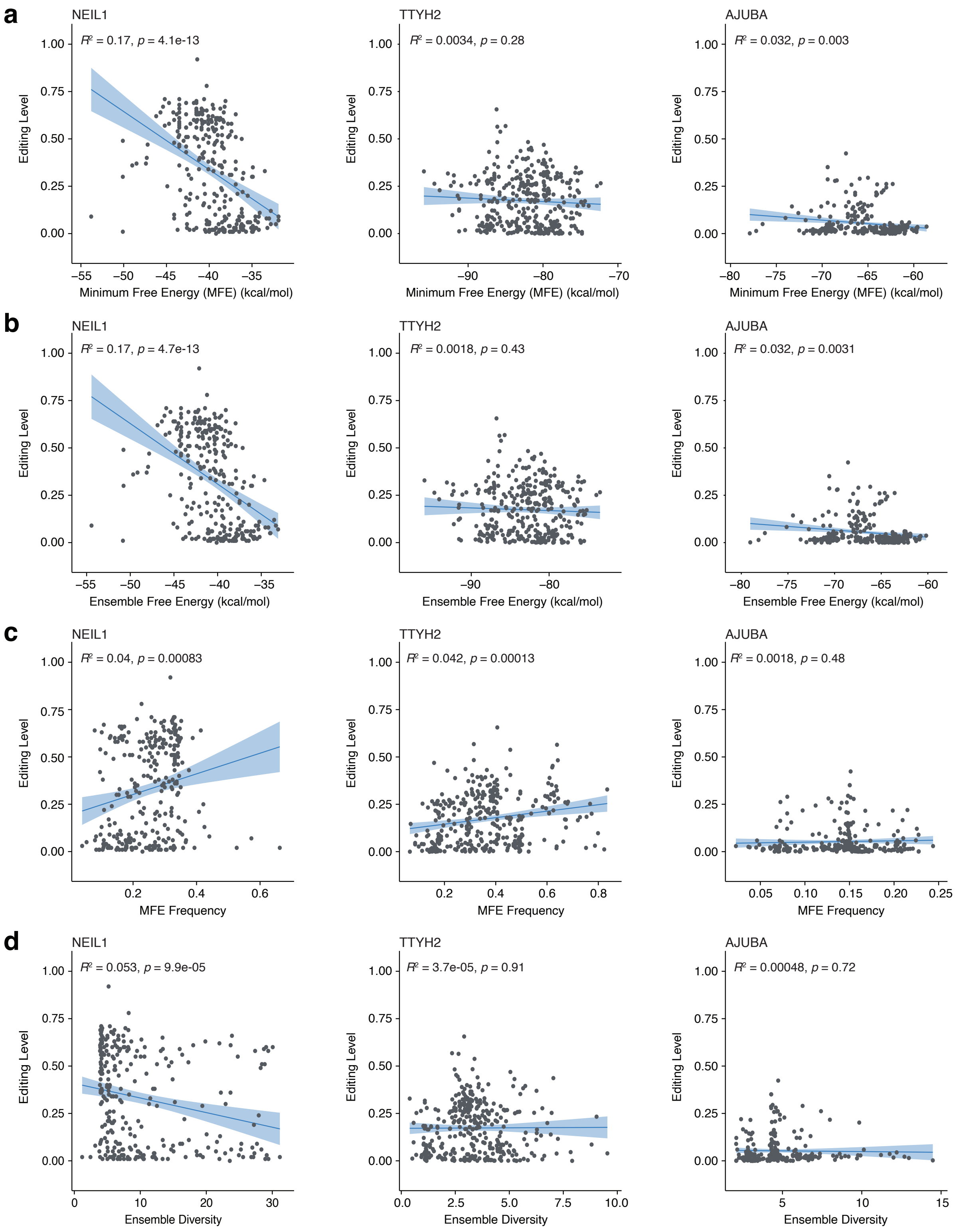

Supplementary Fig. 5 Correlation of selected *cis* regulatory features to editing levels of all variants of NEIL1, TTYH2, and AJUBA. Pearson and spearman correlations were computed between the features in each panel and editing level. Pearson R^2 and Spearman Rho (p) values are provided for each panel. **a** Minimum Free Energy (MFE). **b** Ensemble Free Energy. **c** MFE frequency. **d** Ensemble Diversity. Error band (blue) showing the standard error of the regression.

Supplementary Fig. 6

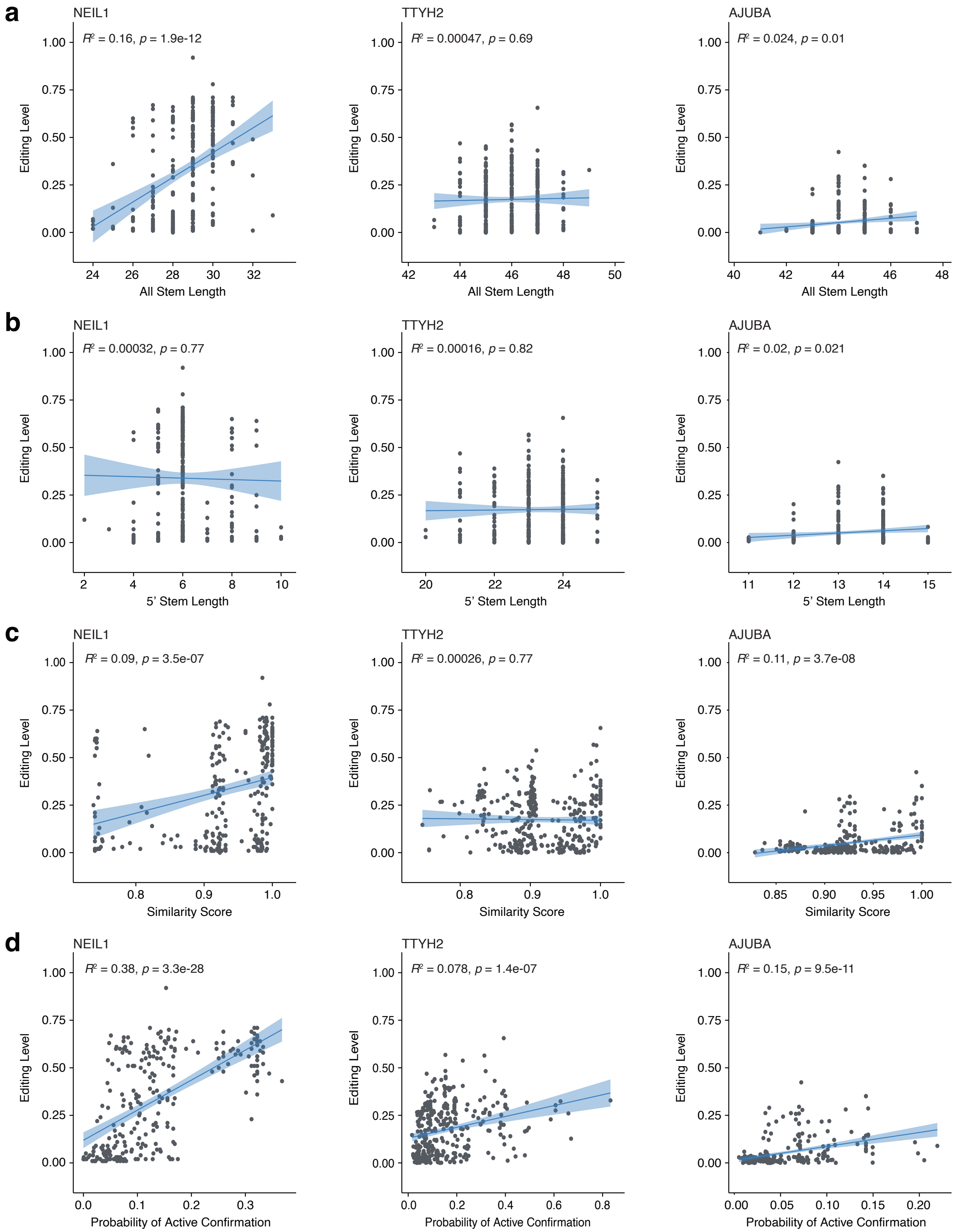

Supplementary Fig. 6 Correlation of selected *cis* regulatory features to editing levels of all variants of NEIL1, TTYH2, and AJUBA (continue). Pearson and spearman correlations were computed between the features in each panel and editing level. Pearson R^2 and Spearman Rho (*p*) values are provided for each

panel. **a.** All Stem Length. **b** 5’ Stem Length. **c** Similarity Score (normalized). **d** Probability of Active Conformation. Error band (blue) showing the standard error of the regression.

Supplementary Fig. 7

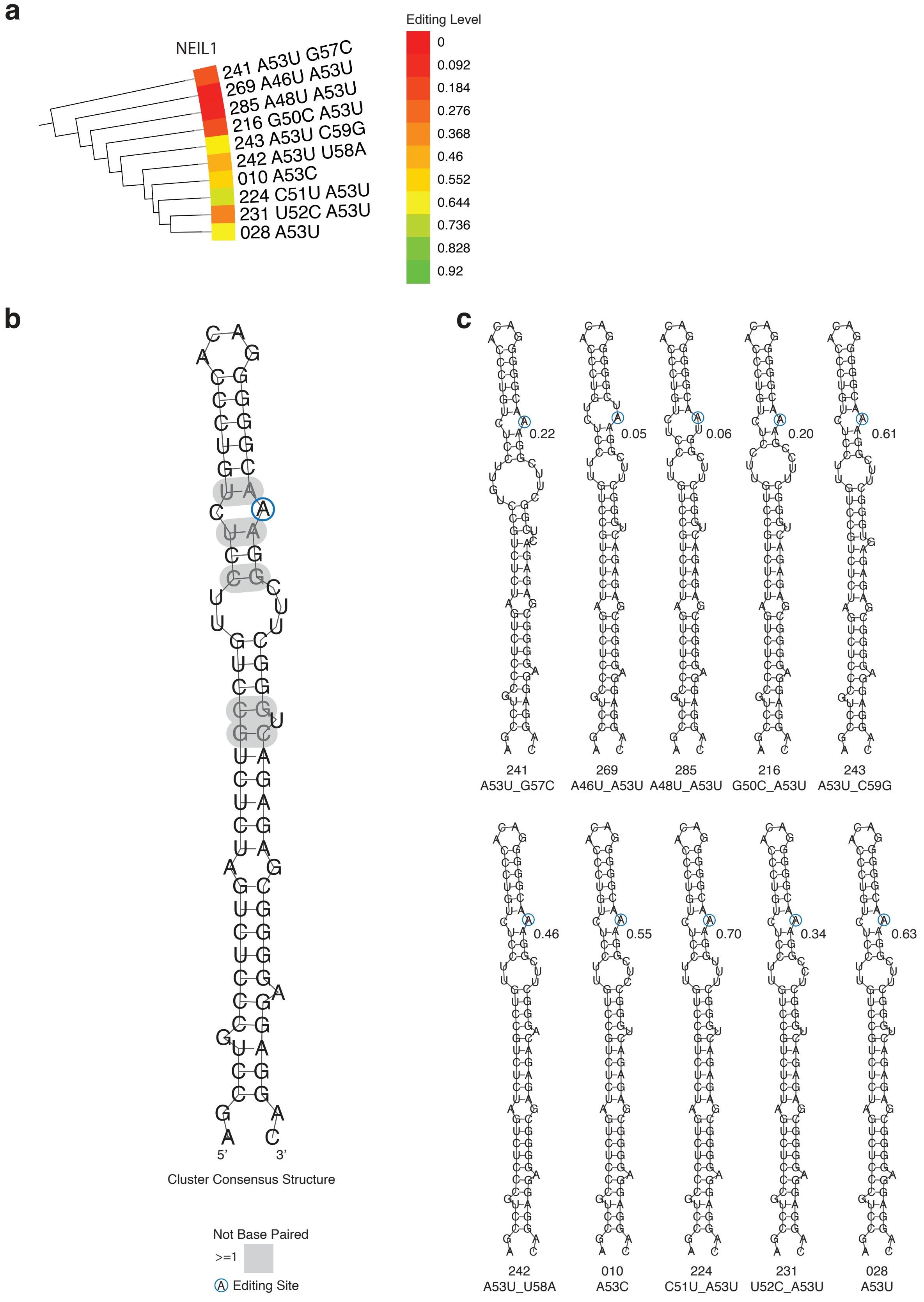

Supplementary Fig. 7 Example of NEIL1 cluster. a Heatmap of one clade from the NEIL1 cluster shown in **Fig. 6**. The editing level of each variant are shown according to the heatmap scale. The sequence and structure corresponding to each RNA ID are listed in **Supplementary Data 2**. **b** The consensus structure of the cluster shown in **a**. **c** the MFE structure of each variant in the cluster. The gray box ("not base paired") in the consensus structure means that there is at least one variant that has a different structure at this position. Blue circle indicates the editing site. The value of editing level is shown as numbers.

Supplementary Fig. 8

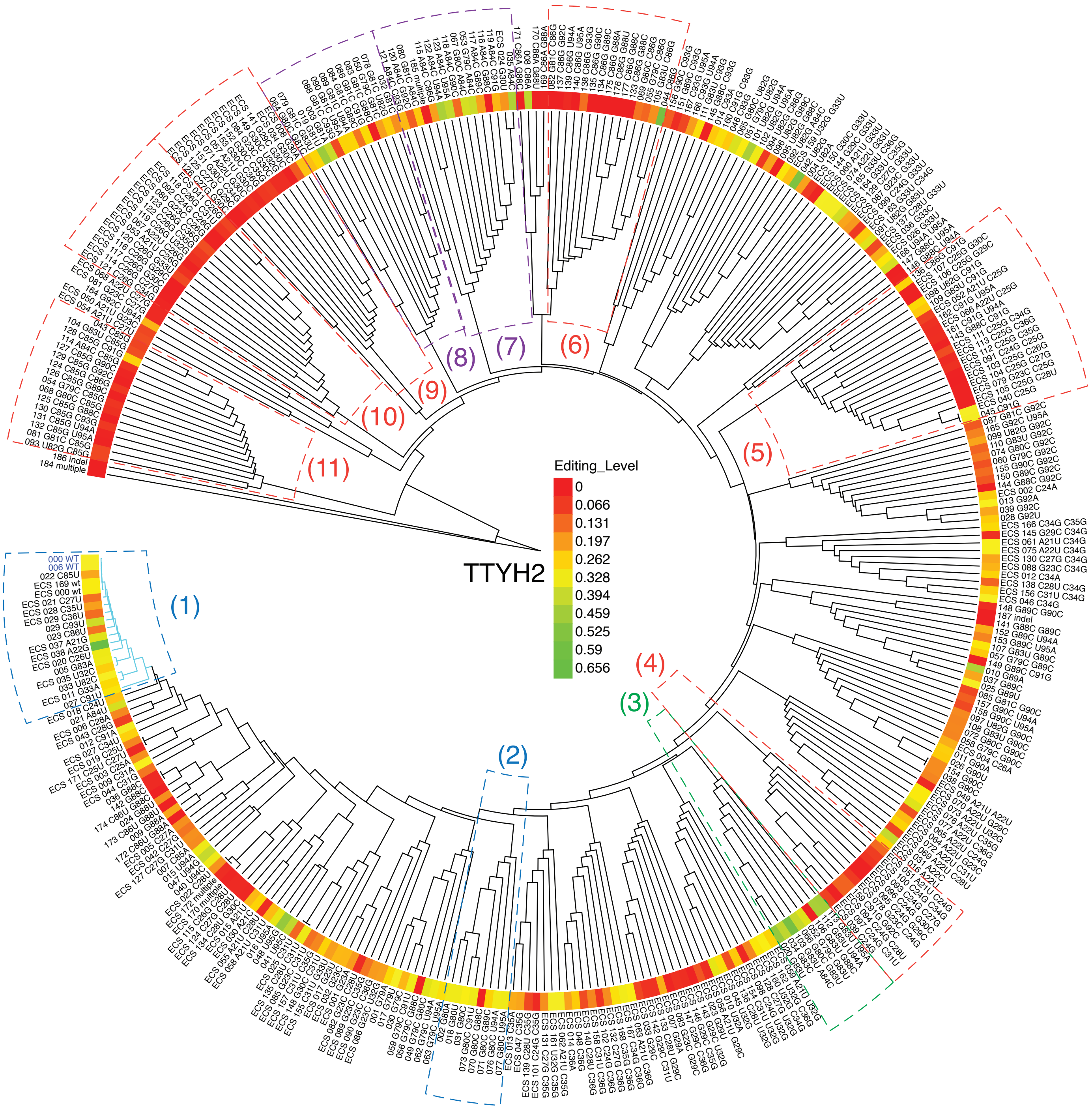

Supplementary Fig. 8 Clustering of TTYH2 with RNA editing levels. Consensus structure of selected clusters are shown in **Supplementary Fig. 9**. The editing level of each variant are shown according to the heatmap scale. The sequence and structure corresponding to each RNA ID are listed in **Supplementary Data 3**.

Supplementary Fig. 9

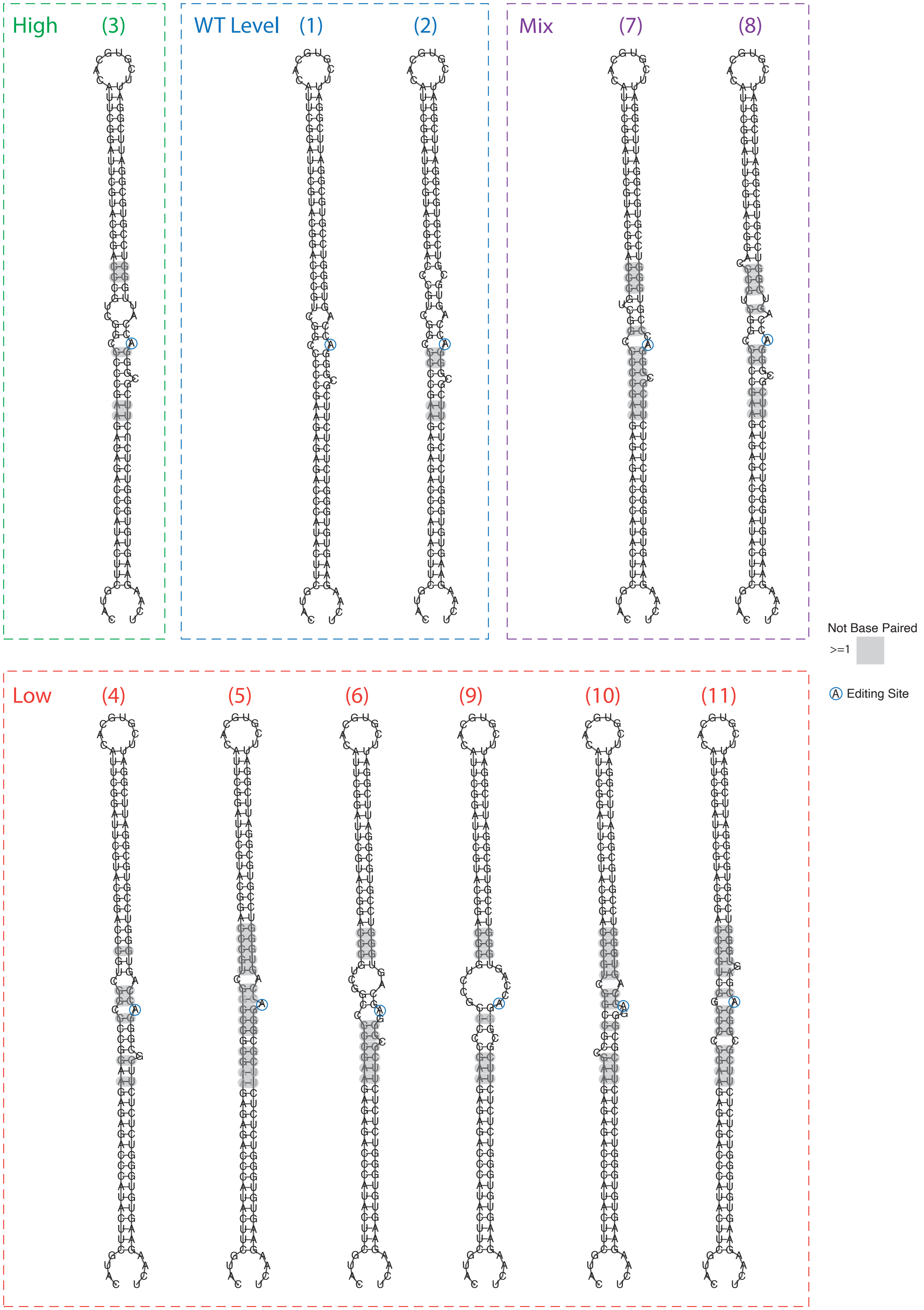

Supplementary Fig. 9 Consensus structures of selected TTYH2 clusters. Editing level for each cluster is shown in **Supplementary Fig. 8**. The editing level of each variant are shown according to the heatmap scale. The sequence and structure corresponding to each RNA ID are listed in **Supplementary Data 3**.

Supplementary Fig. 10

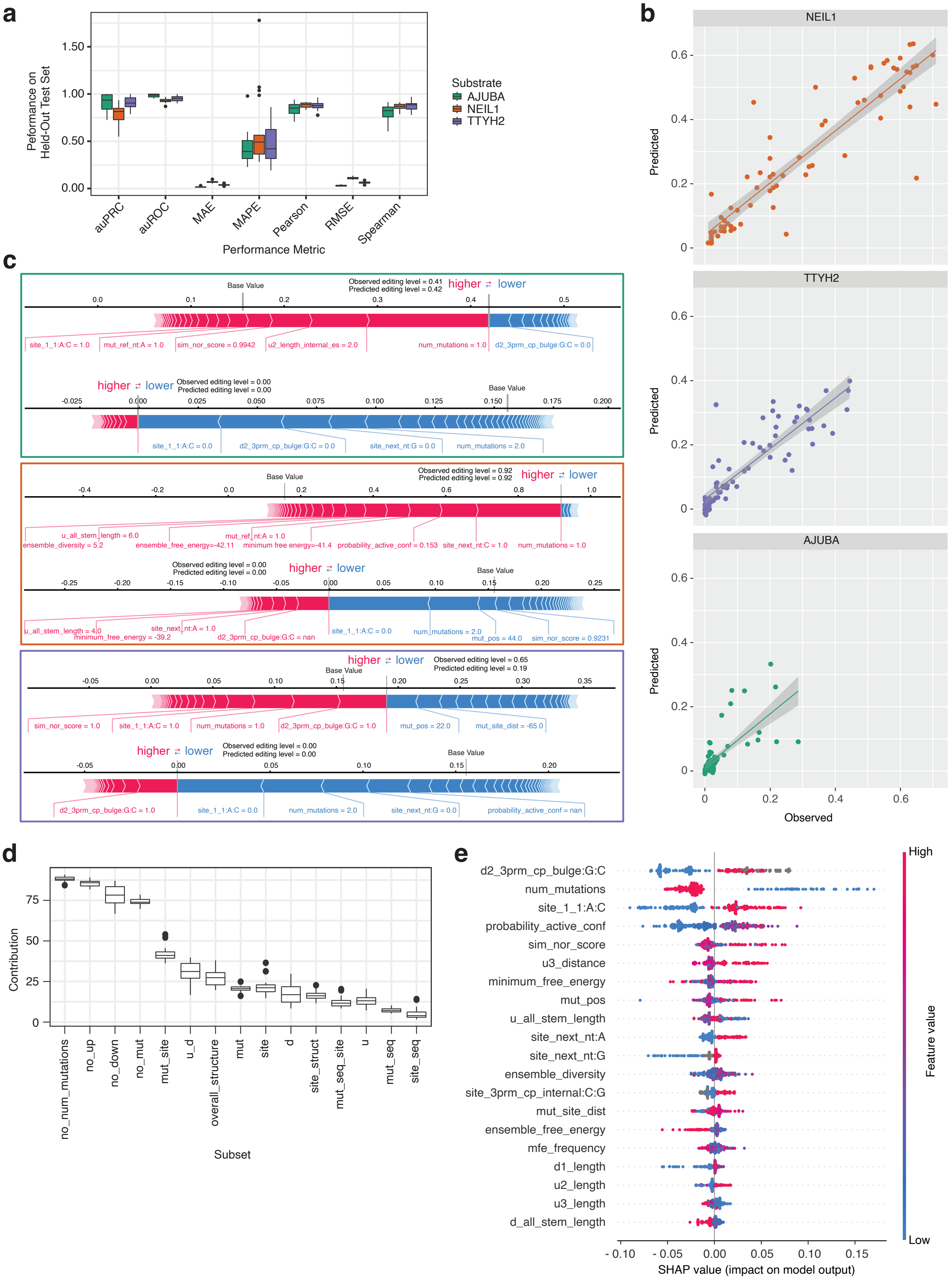

Supplementary Fig. 10 Joint training and testing across substrates. a. Held-out test set performance metrics (spearman, pearson, MAE, MAPE, RMSE, auPRC, auROCe) for model trained jointly on NEIL1 (orange), TTYH2 (purple), and AJUBA (green) substrates. Mean absolute error (MAE), Mean absolute percent error (MAPE), Root mean square error (RMSE), Area under the precision recall curve (auPRC), Area under the receiver operating characteristic (auROC). 20 combinations of training/test/validation splits were generated, error bars indicate the interquartile range of metrics on the test sets for these 20 combinations. Training set size (averaged across the 20 splits): NEIL1=281, TTYH2=409, AJUBA=325. Validation set size: NEIL1=64, TTYH2=91, AJUBA=46. Test set size: NEIL1=72, TTYH2=83, AJUBA=89. Boxplots are presented as median-centered, with the box bounds indicating the 75 percent IQR. Whiskers extend to the data points that fall within the median ± 1.5*IQR. **b.** Test set XGBoost predictions for each substrate from a model strained jointly on NEIL1, TTYH2, and AJUBA. Error bands (in grey) the 95 pointwise confidence bound for the mean predicted value, using linear smoothing. **c.** SHAP values for the variants with highest and lowest editing levels for each substrate. **d** Percent contribution to XGBoost test set predictions from each feature subset. Feature subset composition is defined in **Supplementary Data 1**. Feature contribution is computed for 20 random training/validation set selections from the joint cross-substrate dataset. Boxplots are presented as median-centered, with the box bounds indicating the 75 percent IQR. Whiskers extend to the data points that fall within the median ± 1.5*IQR. **e.** SHAP values for the 20 most important features driving test set predictions. Features are ranked in order of predictive importance from most important (top) to least important (bottom). Error bands (in grey) the 95 pointwise confidence bound for the mean predicted value, using linear smoothing.

Supplementary Fig. 11

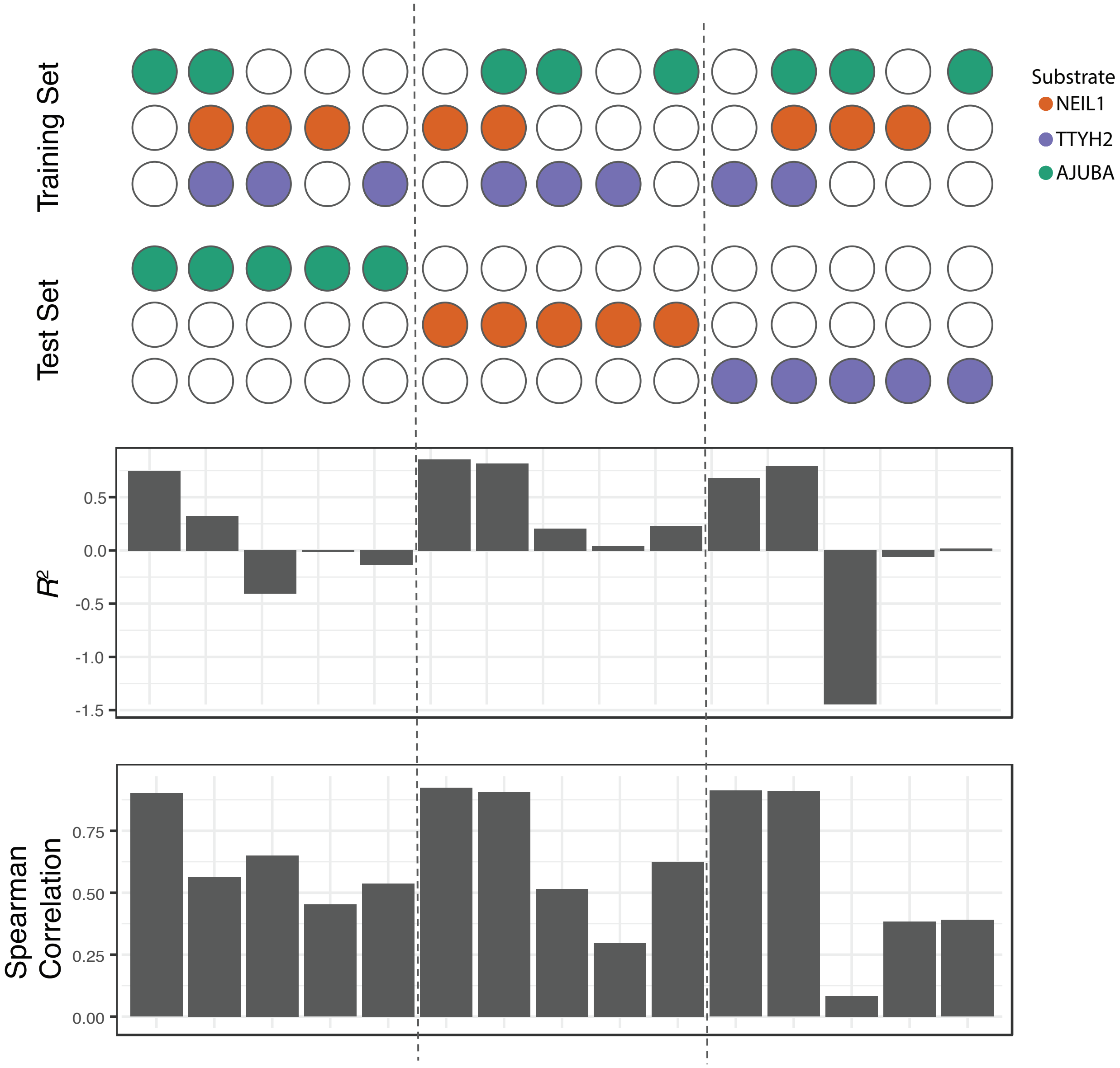

Supplementary Fig. 11 Performance of XGBoost model across training sets. Spearman correlation between observed and predicted ADAR editing levels in the held-out test set, as well as the percent of variance explained in the held-out test set, is illustrated for all combinations of training and test sets examined. Filled-in circles indicate that the particular substrate (NEIL1, orange; TTYH2, purple; AJUBA, green) was included in a given training/test split.
