# Supplementary material for: Learning cis-regulatory principles of ADAR-based RNA editing from CRISPR-mediated mutagenesis
Source: Nat Commun. 2021 Apr 12;12:2165. doi: 10.1038/s41467-021-22489-2 (PMC8041805; doi:10.1038/s41467-021-22489-2)
Supplement: Supplementary file 11 — Description of Additional Supplementary Files [file 41467_2021_22489_MOESM11_ESM.docx]

Description of additional supplementary information

Title: Supplementary Data 1

Description: Feature engineering for machine learning. Sheet 1: Features used with XGBoost for prediction of editing level; Sheet 2: List of each feature sub-group with the features that comprise it. Page 8 of 13

Title: Supplementary Data 2

Description: NEIL1 feature matrix.

Title: Supplementary Data 3

Description: TTYH2 feature matrix.

Title: Supplementary Data 4

Description: AJUBA feature matrix.

Title: Supplementary Data 5

Description: XGBoost prediction performance on the training, validation, and test splits. Performance metrics (% Variance explained, Spearman R, Pearson R, MAE, MAPE, RMSE, auPRC, auROC) are provided for models trained within-substrate, jointly across substrates, and crosssubstrate.

Title: Supplementary Data 6

Description: Sequences of oligonucleotides for CRISPR/Cas9- mediated mutagenesis and in vitro RNA structure probing. Sheet 1: the guide RNA sequence. Sheet 2 and 3: donor oligos for CRISPR. Sheet 4: PCR primers. Sheet 5: oligo templates for in vitro NEIL1 library. Sheet 6: PCR primers to generate DNA for in vitro transcription of NEIL1 RNAs. Sheet 7: oligo templates for in vitro TTYH2-ECS library. Sheet 8: RT and PCR primers to generate sequencing library from in vitro chemical mapping of RNA structure by dimethyl sulphide (DMS). Page 9 of 13

Title: Supplementary Data 7

Description: RNA sequence, mutation name, editing level, editing level Z-score, and computationally predicted RNA secondary structure. Sheet 1: NEIL1. Sheet 2: TTYH2. Sheet 3: AJUBA.

Title: Supplementary Data 8

Description: Normalized SHAP and F1 scores for each feature used to train the substrate specific AJUBA, NEIL1, and TTYH2 models. Relative feature contributions are normalized to sum to 100 for the full set of features. A blank cell indicates that the feature was not used to train a given model due to a lack of training and/or validation examples for that feature/substrate combination. Normalized SHAP scores and normalized F scores from XGBoost are provided.
