# Supplementary material for: Learning cis-regulatory principles of ADAR-based RNA editing from CRISPR-mediated mutagenesis
Source: Nat Commun. 2021 Apr 12;12:2165. doi: 10.1038/s41467-021-22489-2 (PMC8041805; doi:10.1038/s41467-021-22489-2)
Supplement: Supplementary file 12 — Reporting Summary [file 41467_2021_22489_MOESM12_ESM.pdf]

nature research

Corresponding author(s): Jin Billy Li, Anshul Kundaje

Last updated by author(s): Jan 28, 2021

Reporting Summary

Nature Research wishes to improve the reproducibility of the work that we publish. This form provides structure for consistency and transparency in reporting. For further information on Nature Research policies, see our Editorial Policies and the Editorial Policy Checklist.

Statistics

For all statistical analyses, confirm that the following items are present in the figure legend, table legend, main text, or Methods section.

n/a	Confirmed	
☐	☒	The exact sample size (*n*) for each experimental group/condition, given as a discrete number and unit of measurement
☐	☒	A statement on whether measurements were taken from distinct samples or whether the same sample was measured repeatedly
☐	☒	The statistical test(s) used AND whether they are one- or two-sided<br>*Only common tests should be described solely by name; describe more complex techniques in the Methods section.*
☐	☒	A description of all covariates tested
☐	☒	A description of any assumptions or corrections, such as tests of normality and adjustment for multiple comparisons
☐	☒	A full description of the statistical parameters including central tendency (e.g. means) or other basic estimates (e.g. regression coefficient) AND variation (e.g. standard deviation) or associated estimates of uncertainty (e.g. confidence intervals)
☐	☒	For null hypothesis testing, the test statistic (e.g. *F*, *t*, *r*) with confidence intervals, effect sizes, degrees of freedom and *P* value noted<br>*Give P values as exact values whenever suitable.*
☒	☐	For Bayesian analysis, information on the choice of priors and Markov chain Monte Carlo settings
☒	☐	For hierarchical and complex designs, identification of the appropriate level for tests and full reporting of outcomes
☐	☒	Estimates of effect sizes (e.g. Cohen's *d*, Pearson's *r*), indicating how they were calculated

Our web collection on statistics for biologists contains articles on many of the points above.

Software and code

Policy information about availability of computer code

Data collection	https://basespace.illumina.com/ was used to collect sequencing data.
Data analysis	-Bioinformatics codes for RNA editing call are available upon request.<br><br>-Codes for the PREUSS computational pipeline is available on GitHub URL: https://github.com/kundajelab/PREUSS.<br><br>-Software/Code packages used include:<br>General data analysis:<br>R studio 1.3.959<br>Python 3.2<br>GraphPad Prism 6.01<br><br>Sequencing data analysis:<br>GMAP 2018<br>GSNAP 2018<br>FastQC 0.11.8<br><br>RNA structure and modeling<br>RNAfold 2.4.14<br>LocARNA 2.0.0RC8<br>SimTree 1.2.3<br>RNAclust.pl, version 1.3, modified to suit current computing environment<br>Rosetta RNP-denovo

April 2020

Phyre2

RNA structure inference from DMS chemical mapping:
Python 3.2
AfterQC
Cutadapt 1.17
ShapeMapper 2
Biers
MATLAB 2018b

Machine learning:
bpRNA.pl
XGBoost31 Python library (v. 0.81)

For manuscripts utilizing custom algorithms or software that are central to the research but not yet described in published literature, software must be made available to editors and reviewers. We strongly encourage code deposition in a community repository (e.g. GitHub). See the Nature Research guidelines for submitting code & software for further information.

Data

Policy information about availability of data

All manuscripts must include a data availability statement. This statement should provide the following information, where applicable:

- Accession codes, unique identifiers, or web links for publicly available datasets
- A list of figures that have associated raw data
- A description of any restrictions on data availability

The RNA-seq data are deposited in the following repository: Repository/DataBank Accession: GEO; AccessionID: GSE138860. Databank URL: http://www.ncbi.nlm.nih.gov/geo/. The DMS chemical mapping data for in vitro RNA structure inference are deposited in the RNA Mapping Database (RMDB IDs: NEIL1_DMS_0001 to 0021, TTYH2_DMS_0001).

Field-specific reporting

Please select the one below that is the best fit for your research. If you are not sure, read the appropriate sections before making your selection.

☒ Life sciences ☐ Behavioural & social sciences ☐ Ecological, evolutionary & environmental sciences

For a reference copy of the document with all sections, see nature.com/documents/nr-reporting-summary-flat.pdf

Life sciences study design

All studies must disclose on these points even when the disclosure is negative.

Sample size	For experiments, no statistical method was used to pre-determine sample size. All experiments were based on cell biology and/or molecular biology approaches. We made sure to repeat experiments as defined below (Replication) to enable experimental and biological variability to be ascertained. For sequencing runs, each contained on average 10-100 million reads.
Data exclusions	No sequencing run was excluded. Reads were filtered according to specific quality control software depicted in Methods.
Replication	All attempts at replication were successful and detailed included in the manuscript. Briefly, the replicated experiments and analyses are:<br>-Measurements of editing level in HEK293T cells: NEIL1 library 6 biological replicates; TTYH2 library 2 biological replicates; AJUBA library 2 biological replicates.<br>-Measurements of chemical probing of RNA structure in vitro: NEIL1 library 2 biological replicates; TTYH2 library 2 biological replicates.
Randomization	Not applicable to this study. This study does not involve allocation of samples to into groups. We used controls in experiments.
Blinding	Not applicable to this study. This study does not involve allocation of samples to into groups. The investigators were not blinded for collection of experimental data. All data were collected by experimental methods with controls and all samples were treated equally.

Reporting for specific materials, systems and methods

We require information from authors about some types of materials, experimental systems and methods used in many studies. Here, indicate whether each material, system or method listed is relevant to your study. If you are not sure if a list item applies to your research, read the appropriate section before selecting a response.

Materials & experimental systems

n/a	Involved in the study
☒	☐ Antibodies
☐	☒ Eukaryotic cell lines
☒	☐ Palaeontology and archaeology
☒	☐ Animals and other organisms
☒	☐ Human research participants
☒	☐ Clinical data
☒	☐ Dual use research of concern

Methods

n/a	Involved in the study
☒	☐ ChIP-seq
☒	☐ Flow cytometry
☒	☐ MRI-based neuroimaging

Eukaryotic cell lines

Policy information about cell lines

Cell line source(s)	HEK293T cell lines from ATCC
Authentication	None of the cell lines were authenticated
Mycoplasma contamination	Cell lines were not tested for mycoplasma contamination
Commonly misidentified lines (See ICLAC register)	No commonly misidentified cell lines were used in the study
